# Supplementary material for: The Synergistic Effect of Lemongrass Essential Oil and Flometoquin, Flonicamid, and Sulfoxaflor on Bemisia tabaci (Genn.) (Hemiptera: Aleyrodidae): Insights into Toxicity, Biochemical Impact, and Molecular Docking
Source: Insects. 2024 Apr 24;15(5):302. doi: 10.3390/insects15050302 (PMC11122410; doi:10.3390/insects15050302)
Supplement: Supplementary file 1 [file insects-15-00302-s001.zip › insects-2967128-supplementary.pdf]

Table S1. The chemical compounds of lemongrass (*Cymbopogon citratus)* essential oil, as identified by Moustafa et al. (2021) and Moustafa et al. (2023).

RT	Area %	Compound name
7.97	0.83	Isoneral
8.37	1.49	Isogeranial
8.60	0.71	Dihydronopol
9.77	35.00	β-Citral (neral)
10.47	35.91	α-Citral (Geranial (Citral A)
10.61	3.58	*trans*-Verbenol
10.71	0.91	Epoxy-linalooloxide
10.97	1.45	Geranyl vinyl ether
11.26	7.84	Nerylacetal
11.76	9.08	5-Octyldihydro-2(3H)-furanone
12.69	1.24	Geraniol acetate
13.74	1.24	(Z,E)-α-farnesene
16.77	0.72	β-Caryophyllene epoxide

RT (retention time)

Table S2. Analysis of variance (ANOVA) showing the differences of acetylcholine esterase (AchE), Cytochrome P450 (P450), α-esterase, Glutathione S transferase (GST), enzymes specific activity, between lemongrass essential oil, flometoquin, flonicamid, and sulfoxaflor, at their LC_{50} values, Compared to untreated check of *Bemisia tabaci* adults, at 48 h post-treatment.

Enzyme	*F(df)**	*P*
**AchE**	4.48 (4,10)	0.02
**P450**	3.74 (4,10)	0.04
**α-esterase**	4.84 (4,10)	0.02
**GST**	1.62 (4,10)	0.24

*df= degree of freedom

Table S3. Analysis of variance (ANOVA) showing the differences of acetylcholine esterase (AchE), Cytochrome P450 (P450), α-esterase, Glutathione S transferase (GST), enzymes specific activity, between the individual treatments of lemongrass essential oil, flometoquin, flonicamid, and sulfoxaflor, at their LC25 values, and the binary mixture of lemongrass essential oil, at its LC25, with flometoquin, flonicamid, or sulfoxaflor, compared to untreated check of *Bemisia tabaci* adults, at 48 h post-treatment.

**Mixtures**	**AchE**		**P450**		**α-esterase**		**GST**	
	F(df)	*P*	*F (df)*	*P*	*F(df)*	*P*	*F(df)*	*P*
Lemongrass/ flometoquin	7.39(3, 8)	0.01	0.10(3, 8)	0.96	6.45 (3, 8)	0.01	4.05(3, 8)	0.05
Lemongrass/ flonicamid	4.19(3, 8)	0.04	8.23(3,8)	0.01	2.66 (3, 8)	0.12	4.47(3, 8)	0.04
Lemongrass/ sulfoxaflor	6.48(3, 8)	0.015	4.74(3,8)	0.03	1.39 (3, 8)	0.31	2.53(3, 8)	0.13
